# Impact of COVID-19 Restrictions on Incidence of Enteropathogenic Bacteria, Virus, and Parasites in Denmark: A National, Register-Based Study

**DOI:** 10.3390/microorganisms12061224

**Published:** 2024-06-18

**Authors:** Kumanan Rune Nanthan, Eva Plantener, John Coia, Jørgen Engberg, Leif Percival Andersen, Ea Marmolin, Gitte Nyvang Hartmeyer, Hans Linde Nielsen, Christen Rune Stensvold, Anne Line Engsbro, Bente Olesen, Lars Lemming, Ming Chen

**Affiliations:** 1 Department of Clinical Microbiology, University Hospital of Southern Denmark, 6200 Aabenraa, Denmark; 2 Department of Clinical Microbiology, University Hospital of Southern Denmark, 6700 Esbjerg, Denmark; 3 Department of Clinical Microbiology, Zealand University Hospital Koege, 4600 Koege, Denmark; 4 Department of Clinical Microbiology, Copenhagen University Hospital (Rigshospitalet), 2100 Copenhagen, Denmark; 5 Department of Clinical Microbiology, University Hospital of Southern Denmark, 7100 Vejle, Denmark; 6 Department of Clinical Microbiology, Odense University Hospital, 5000 Odense, Denmark; 7 Department of Clinical Microbiology, Aalborg University Hospital, 9000 Aalborg, Denmark; 8 Department of Bacteria, Parasites & Fungi, Statens Serum Institut, 2300 Copenhagen, Denmark; 9 Department of Clinical Microbiology, University Hospital of Hvidovre, 2650 Copenhagen, Denmark; 10 Department of Clinical Microbiology, University Hospital of Herlev, 2730 Copenhagen, Denmark; 11 Department of Clinical Microbiology, Aarhus University Hospital, 8200 Aarhus, Denmark

**Keywords:** SARS-CoV-2, enteropathogenic bacteria, virus, parasites

## Abstract

Diarrheal diseases caused by enteric pathogens are a significant public health concern. It is widely considered that close contact between persons, poor hygiene, and consumption of contaminated food are the primary causes of gastroenteritis. Clinical microbiology laboratory observations indicate that the incidence of enteropathogenic microorganisms may have been reduced in Denmark during the COVID-19 pandemic. All Departments of Clinical Microbiology in Denmark provided data on the monthly incidence of *Salmonella* spp., *Escherichia coli*, *Campylobacter* spp., *Clostridioides difficile*, Norovirus GI+GII, *Giardia duodenalis*, and *Cryptosporidium* from March 2018 to February 2021. The data were divided into three periods as follows: Control Period 1 (March 2018 to February 2019); Control Period 2 (March 2019 to February 2020); and the Restriction (pandemic) Period (March 2020 to February 2021). The incidences of pathogenic *Salmonella* spp.-, *Escherichia coli*-, and *Campylobacter* spp.-positive samples decreased by 57.3%, 48.1%, and 32.9%, respectively, during the restriction period. No decrease in *C. difficile* was observed. Norovirus GI+GII-positive samples decreased by 85.6%. *Giardia duodenalis*-positive samples decreased by 66.2%. *Cryptosporidium* species decreased by 59.6%. This study demonstrates a clear decrease in the incidence of enteropathogenic bacteria (except for *C. difficile*), viruses, and parasites during the SARS-CoV-2 restriction period in Denmark.

## 1. Introduction

In January 2020, the World Health Organization declared severe acute respiratory syndrome coronavirus 2 (SARS-CoV-2) a public health emergency. The disease spread rapidly, infecting more than 100 million people globally and resulting in more than 2 million deaths within the first year [1]. Governments worldwide, including the Danish government, reacted quickly and implemented various restrictions to limit the spread and transmission of the virus [2]. These non-pharmaceutical interventions (NPIs) included social distancing, a focus on improved hygiene such as hand washing and sanitizing, travel restrictions, lockdown of schools and daycare centers, closure of businesses and workplaces, and restrictions on public gatherings.

During peak COVID-19 incidence periods, it was observed that healthcare-seeking behaviors changed significantly. Non-COVID-related hospital admission rates dropped markedly as healthcare providers deprioritized elective healthcare and transitioned to virtual consultations to mitigate the risk of system collapse and prevent nosocomial transmission of COVID-19 [3,4]. Studies have also pointed out that during the COVID-19 pandemic, the incidence of other urgent medical conditions such as stroke, surgical emergencies, cancer, and cardiac emergencies decreased because of behavioral changes among patients, resulting in worse patient outcomes and higher mortality rates among patients with these diagnoses [3,5,6,7]. 

Conversely, an increasing number of studies suggest a decline in infectious diseases other than COVID-19 during the peak of the pandemic. This decreased transmission of communicable diseases, likely mitigated by measures such as social distancing and improved hand hygiene, reflects the NPIs implemented during the COVID-19 pandemic [8,9,10,11,12,13]. 

Clinical observations in Denmark indicated a decrease in acute gastroenteritis (AGE) cases during the COVID-19 restriction period. AGE, characterized by the sudden onset of diarrhea with or without vomiting, generally poses a significant burden of morbidity, mortality, and disease [14]. Studies have reported varying AGE incidence rates worldwide, ranging from 0.274 episodes per person per year in the U.K. [15] to as high as 1.4 in Denmark and the United States [16,17]. Norovirus and rotavirus are common viral causes, while diarrheagenic *Escherichia coli*, *Salmonella* spp., and *Campylobacter* spp. are the bacteria most often involved [18]. Protozoal cases of AGE include *Giardia duodenalis* and *Cryptosporidium* [19]. The typical transmission routes—fecal–oral transmission through contaminated food and water and direct contact with infected individuals, along with international travel to regions with higher gastrointestinal infection ratios—were indirectly affected during the COVID-19 restrictions because of population behavioral changes and travel restrictions [20]. 

A couple of studies have been published describing a decrease in the incidence of AGE in children during COVID-19-associated restrictions [21,22,23,24]. These studies mostly focused on the AGE incidence rates in children, with other population studies only focusing on the first months of the COVID-19 outbreak. Moreover, certain studies solely report on clinical incidence without including data on the causative microorganisms of AGE. To our knowledge, no study to date has utilized nationwide data from Denmark to assess the impact of the COVID-19 restrictions on AGE-causative agents.

This study aims to examine the impact of SARS-CoV-2 restrictions implemented in Denmark on the incidence of diagnosed AGE across all of Denmark, with a comparison of AGE-causative agents detected before and during the restrictions.

## 2. Materials and Methods

### 2.1. Approvals

This retrospective register study involved the collection of data after patients had undergone examinations and provided stool samples. Obtaining permission for inclusion from individual patients was deemed unnecessary, as this study did not influence further patient treatment. The Danish Data Protection Agency approved this study (j.nr 2021-522-0329). This study was also approved by the Region of Southern Denmark (journal number 21/34765).

### 2.2. Outcome

The primary objective of this study was to compare the incidence of selected enteropathogenic microorganisms in Denmark before and during the SARS-CoV-2 restriction periods, providing monthly data on the incidence of specific agents causing AGE to understand which microorganisms were most affected by the restrictions.

### 2.3. Study Design

This nationwide multicenter retrospective register study collected data from all 10 Clinical Microbiological Departments in Denmark and the Danish Statens Serum Institut. Positive stool samples analyzed for gastrointestinal (GI)-associated pathogens between March 2018 and February 2021 were extracted from two respective laboratory information systems, MADS and WWBakt (Autonik AB, Sweden). Because of differences in routine procedures among the departments participating in this study, it was difficult to obtain the total number of all stool samples analyzed. It is possible that one patient had multiple positive samples registered. Pathogens included in this study were diarrheagenic *Escherichia coli* (enteropathogenic *E. coli* (EPEC), enterohemorrhagic (Shiga toxin-producing) *E. coli* (EHEC/STEC), enteroaggregative *E. coli* (EAEC), enterotoxigenic *E. coli* (ETEC), and enteroinvasive *E. coli* (EIEC)), *Campylobacter jejuni*/*coli*/*upsaliensis*/*fetus*, *Salmonella typhi*/*paratyphi*, zoonotic *Salmonella* spp., *Clostridioides difficile*, Norovirus GI+GII, *Giardia duodenalis*/*lamblia*/*intestinalis*, and *Cryptosporidium parvum*/*hominis*/spp.

### 2.4. Infection Control Measures in Denmark

The Danish Ministry of Health implemented the first initiatives to prevent the spread of SARS-CoV-2 on 6 March 2020, by recommending a limitation of large gatherings to 1000 people. Subsequently, on 11 March 2020, the Prime Minister of Denmark introduced the first lockdown, which involved the closure of all day-care centers, schools, and the majority of workplaces. This lockdown persisted until 15 April 2020, when day-care centers and school classes up to 6th grade reopened with preventive measures in place, including social distancing, frequent SARS-CoV-2 testing, and continued societal restrictions. A gradual reopening of society occurred from 9 June to 30 September 2020. However, because of a rise in infection rates, a gradual regional lockdown ensued from 1 October to 15 December 2020, resulting in a second nationwide lockdown from 16 December 2020 to 27 January 2021. Denmark commenced a gradual reopening on 1 March 2021, easing restrictions until a complete return to normal living was achieved on 10 September 2021.

### 2.5. Dataset and Time Periods

The dataset was segmented into a pre-pandemic phase (Control Period 1: March 2018 to February 2019; Control Period 2: March 2019 to February 2020) compared with the pandemic phase (Restriction Period) defined as 1 March 2020 to 28 February 2021. Each period spanned twelve months to easily compare the two control periods pr. month.

### 2.6. Statistical Analysis

Data on AGE causative microorganisms was extracted from the MADS and WWBakt databases. Monthly positive stool samples for each pathogen were summarized and compared with the same months in the following years. A paired two-tailed *t* test was used for analysis of the data. *p*-values of <0.05 were considered significant.

## 3. Results

In total, 71,049 positive stool samples were identified in this study during the study period, with 25,061 in Control Period 1, 27,882 in Control Period 2, and 18,106 in the Restriction Period. During the first two control periods from 1 March 2018 to 29 February 2020, an average of 2206.2 positive stool samples per month was identified (2088.4 in Control Period 1, 2324.0 in Control Period 2) This number decreased significantly to 1508.8 during the Restriction (pandemic) Period, marking a 31.6% reduction (*p* < 0.05). Table 1 summarizes positive bacterial, viral, and parasitical stool samples.

**Figure 1:**
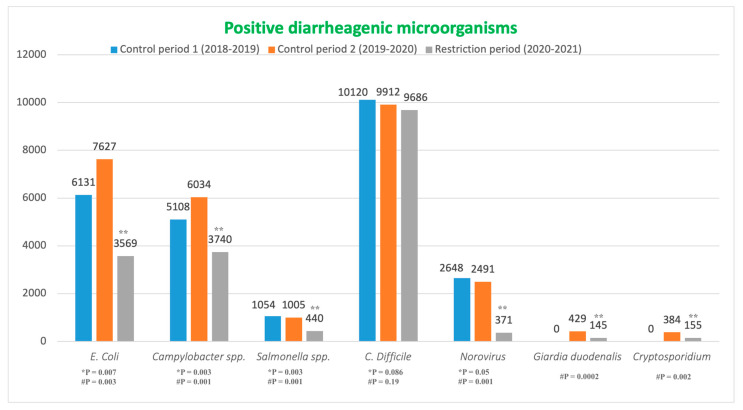
Comparison of positive diarrheagenic *E. coli*, *Campylobacter* spp., *Salmonella* spp., *C. difficile*, Norovirus, *G. duodenalis* and *Cryptosporidium* in Denmark before and during the SARS-CoV-2 Restriction Period. * *p*-values comparing Control Period 1 vs. the Restriction Period. # *p*-values comparing Control Period 2 vs. the Restriction Period. ** Indicates statistical difference.

*E. coli:* During the study period, a total of 17,327 stool samples were positive for diarrheagenic *E. coli*. In Control Period 1, there were 6131 stool samples positive for diarrheagenic *E. coli* (510.9 pr. month on average). In Control Period 2 the total was 7627 (635.6 positive stool samples pr. month on average). During the Restriction Period, the total decreased to 3569 (297.4 pr. month on average), corresponding to a 48.1% decrease when compared with the average of the control periods (Figure 1).

*Campylobacter* spp.: In total, 14,888 stool samples were positive for *Campylobacter* spp. during the study period. Overall, 5108 (425.7 pr. month) and 6034 (503.3 pr. month) positive stool samples were identified during the first and second control periods, respectively. A 32.9% decrease was observed during the Restriction Period, with a total of 3740 positive samples (Figure 1).

*Salmonella* spp.: During this study, there were 2499 positive stool samples for *Salmonella* spp. Control Period 1 had a total of 1054 positive samples (87.8 pr. month), while this number was 1005 during Control Period 2 (83.8 pr. month). However, during the Restriction Period, there was a significant decrease of 57.3%, resulting in 440 positive samples (36.7 pr. month) (Figure 1).

*C. difficile:* A total of 29,718 stool samples were positive for *C. difficile* during this study. Control Period 1 saw a total of 10,120 stool samples (843.3 pr. month), while this number was 9912 during Control Period 2 (826.0 pr. month). In the Restriction Period, 9686 positive samples were identified (807.2 pr. month), which meant a decrease of 3.3% compared with the average of the control periods (Figure 1).

Norovirus: There were 5510 stool samples identified for Norovirus throughout the study period. During the first two control periods, a total of 2648 (220.7 pr. month) and 2491 (207.6 pr. month) positive samples for Norovirus GI+GII were identified, respectively. A substantial 85.6% reduction in positive Norovirus samples was observed during the Restriction Period, with only 371 positive stool samples (30.9 pr. month) (Figure 1).

*G. duodenalis*: In total, 574 stool samples positive for *G. duodenalis* were observed. During Control Period 2, there was a total of 429 samples (35.8 pr. month), while the total decreased to 145 during the Restriction Period (12.1 pr. month), corresponding to a decrease of 66.2% (Figure 1).

*Cryptosporidium* spp: In total, 539 stool samples positive for cryptosporidium were observed. During Control Period 2, there was a total of 384 samples (32.0 pr. month), while this number decreased to 155 during the Restriction Period (12.9 pr. month), corresponding to a decrease of 59.6% (Figure 1).

## 4. Discussion

This study found a 31.6% decrease in AGE-causing positive stool samples during the SARS-CoV-2 Restriction Period compared with the previous two years in Denmark. This decline has been reported in similar studies during the SARS-CoV-2 Restriction Period. Plantener et al. found a 32.2% decrease in AGE-causing positive stool samples during the SARS-CoV-2 Restriction Period; however, their study only reported data from children under 18 and only involved three regional hospitals in Southern Denmark [25]. Eigner et al., from Germany, reported that among hospitalized patients, a significant decrease in positive stool samples for Norovirus (NoV) was observed after the introduction of SARS-CoV-2 restrictions. They highlighted the normal seasonal variation in NoV-positive tests and reported the lowest proportion of NoV-positive tests to be in July (4.3%) and August (4.8%) 2019. In 2020, the percentage of NoV-positive specimens decreased sharply after the introduction of SARS-CoV-2 restrictions, reaching nearly 0% NoV-positive samples by May 2020 and remaining at 1–2% for the rest of the year [26]. An Israel-based study by Bassal et al. reported a relative risk reduction of 86.6% for Shigellosis, 33.0% for Salmonellosis, and 30.0% for Campylobacteriosis [12]. In the U.K., Love et al. reported a decrease of 34% in laboratory-confirmed GI outbreaks during the first seven months of 2020 compared with the pre-pandemic 5-year period [22]. In Norway, Knudsen et al. reported a significant reduction in positive stool samples in children with adenovirus and Norovirus during the pandemic months compared with the pre-pandemic months [21].

Interestingly, the timing and proportion of SARS-CoV-2 restrictions varied between different nations’ strategies [25]. However, our study indicates that the core principles of social distancing, travel restrictions, and behavioral changes contributed to significant reductions in GI infections. Studies have shown that during the SARS-CoV-2 pandemic, healthcare-seeking behavior changed drastically. People were more reluctant to seek hospitals and doctors for both milder and emergency illnesses [4,6,7,27,28]. This reluctance could have contributed to the decrease in monthly positive stool samples, with AGE patients choosing to stay at home rather than seeking medical attention and having stool samples assessed in a laboratory. In England, syndromic indicators for non-infectious GI conditions showed a rapid return to baseline levels during the lockdown, while GI infections showed a more modest increase [22]. This indicates that adherence to hygiene and social distancing measures issued to prevent widespread SARS-CoV-2 infection might have had protective effects on GI infections depending on the pathogen. Norovirus had the greatest reduction in this study, with an 85.6% decrease after the implementation of restrictions. This indicates that social distancing measures and hygiene had a remarkable impact on person-to-person transmitted pathogens, such as Norovirus [29].

Bacterial pathogens, such as *Salmonella* spp., *E. coli*, and *Campylobacter* spp. showed lesser reductions of 57.3%, 48.1%, and 32.9% respectively. *Salmonella* spp. and *E. coli* are also associated with person-to-person and fecal–oral transmission and thus are also likely to have been affected by hygiene and social distancing measures [30,31]. *Campylobacter* spp. is usually transmitted through incorrect food preparation [32]. The restrictions involving the closure of restaurants and shopping malls combined with reduced risk of cross-contamination due to improved hygiene might have contributed to the reduction in *Campylobacter* spp. The reduction in *Salmonella* spp. and *G. duodenalis* is likely to be related to SARS-CoV-2 travel restrictions, which involved a recommendation to avoid all non-essential travel to foreign countries [33]. *C. difficile* maintained its prevalence before and during the SARS-CoV-2 pandemic. This is likely because this infection is associated with antibiotic usage and is not directly related to personal hygiene. 

This study’s strength lies in its large sample size sourced from reliable national electronic databases. The control periods were restricted to two years prior to the implementation of SARS-CoV-2 restrictions, and there were no follow-up data after the restrictions were lifted. Future studies should include post-pandemic data as they could provide insight into an eventual post-pandemic hyperinfection due to waning mucosal immunity. Data on the number of total assessed samples are not presented in this study. The lack of assessed samples is a risk of bias as the results might be affected by a reduced number of assessed samples because of changes in healthcare-seeking behavior during the pandemic. No significant differences (*p* > 0.05) were found between control groups 1 and 2, indicating that our control periods were not subject to any decisive spikes or outbreaks that might have inflated the number of positive stool samples.

## 5. Conclusions

This study indicates that SARS-CoV-2 infection control measures such as social distancing, improved hygiene including handwashing with soap or using alcohol-based hand sanitizers, and limited foreign travel might have contributed as non-pharmaceutical interventions to reduce the prevalence of GI infection-causing pathogens. Future studies should examine compliance with restrictions and how prevalence changed as society gradually phased out SARS-CoV-2 restrictions and returned to normal life.

## Figures and Tables

**Table 1:** Incidence of enteropathogenic bacteria, viruses, and parasites. *p*-values comparing control-period 2 vs. restriction period.

Incidence of Enteropathogenic Microorganisms					
Time Period	Control Period 1	Control Period 2	Restriction Period	Total	*p*-Value
Bacteria	22,413	24,578	17,435	64,426	0.012
Viruses	2648	2491	371	5510	<0.001
Parasites	-	813	300	1113	<0.001
Total	25,061	27,882	18,106	71,049	

## Data Availability

The raw data supporting the conclusions of this article will be made available by the authors on request.
